# Efficacy of factory-treated and dip-it-yourself long lasting insecticide-treated bednets against cutaneous leishmaniasis vectors in the sub-Andean region of Colombia: results after two years of use

**DOI:** 10.1590/0074-02760190431

**Published:** 2020-09-11

**Authors:** Erika Santamaría, Olga Lucía Cabrera, Raúl Hernando Pardo

**Affiliations:** Instituto Nacional de Salud, Grupo de Entomología, Bogotá, Colombia

**Keywords:** sand flies, Lutzomyia, prevention and control, lambda-cyhalothrin, deltamethrin

## Abstract

**BACKGROUND:**

Long lasting insecticide-treated nets (LLINs) may be effective for vector control of cutaneous leishmaniasis (CL). Their efficacy, however, has not been sufficiently evaluated.

**OBJECTIVE:**

To evaluate the large-scale efficacy of LLINs on *Lutzomyia longiflocosa* entomological parameters up to two years post-intervention in the sub-Andean region of Colombia.

**METHODS:**

A matched-triplet cluster-randomised study of 21 rural settlements, matched by pre-intervention *L. longiflocosa* indoor density was used to compare three interventions: dip it yourself (DIY) lambda-cyhalothrin LLIN, deltamethrin LLIN, and untreated nets (control). Sand fly indoor density, feeding success, and parity were recorded using CDC light trap collections at 1, 6, 12, and 24 months post-intervention.

**FINDINGS:**

Both LLINs reduced significantly (74-76%) the indoor density and the proportion of fully engorged sand flies up to two years post-intervention without differences between them. Residual lethal effects of both LLINs and the use of all nets remained high throughout the two-year evaluation period.

**CONCLUSIONS:**

Both LLINs demonstrated high efficacy against *L. longiflocosa* indoors. Therefore, the deployment of these LLINs could have a significant impact on the reduction of CL transmission in the sub-Andean region. The DIY lambda-cyhalothrin kit may be used to convert untreated nets to LLINs increasing coverage.

Cutaneous leishmaniasis (CL) is endemic and the predominant form of leishmaniasis in the Andean region (Venezuela, Colombia, Ecuador, Perú and Bolivia) where 86,000 to 141,000 cases are estimated to occur each year.^1^ In Colombia, where 95% of reported cases correspond to CL with a reported median annual incidence above 9,000 cases (2011-2016), all leishmaniases [CL, mucocutaneous, and visceral (VL)] are important vector borne diseases, and the country is one of the ten countries which contribute with most (75%) of CL cases worldwide.^1^ The country has 45% (9/20) of *Leishmania* species proven to be human etiologic agents of leishmaniases^2^ and 15% (15/100) of the proven and suspected sand fly vectors worldwide. CL is present principally in rural areas of the sub-Andean region (altitudinal range in the Andes from 1,000-2,400 m a.s.l.) where a significant proportion of transmission occurs indoors and in the peridomestic environment similar to other countries in the region.^3^

*Lutzomyia longiflocosa* Osorno-Mesa, Morales, Osorno & Hoyos 1970 is one of the primary vectors of CL in Colombia.^4^ This sand fly inhabits sub-Andean fragmented forests and habitats with similar structure (e.g., traditional coffee plantations). *L. longiflocosa* is mainly a nocturnal biter with preference for feeding inside houses (endophagy); peak biting activity occurs from 22:00 to 00:00 h with lower activity at dusk and dawn.^5^ In locations with the highest sand fly densities, peak biting activities have been observed between 2:00 h to 6:00 h (Unpublished observations by the authors).

Vector control remains one of the most important components of leishmaniases control. The principal methods are indoor residual spraying (IRS), untreated mosquito nets (bednets), and insecticide-treated nets. The latter method includes: (a) conventional insecticide-treated nets, CITNs (nets are manually treated by dipping them in insecticide with required annual retreatment to ensure insecticidal effect); and (b) long lasting insecticide-treated nets, LLINs (factory-treated nets formulated to retain their insecticidal effect for at least 20 washes and three years of use under field conditions).^6^ Therefore, LLINs should provide longer protection for users and be more cost effective than CITNs. In Colombia, leishmaniasis control targets adult sand flies, using IRS, LLINs and CITNs as part of an integrated disease management approach. In practice however, deployment of these strategies is usually reactive, targeting sites/houses where transmission has occurred. Unfortunately, in the context of national programs, their impact on entomological or epidemiological endpoints are not assessed.

Although LLINs have been used globally against leishmaniases for more than a decade, research studies on their impact is principally on Old Word VL vectors (*Phlebotomus* spp.) measuring only entomological endpoints. These studies show significant reductions in sand fly indoor densities, but for short follow-up periods up to one year.^7^ ^,^ ^8^ ^,^ ^9^ A single study from Iran showed that permethrin LLINs significantly reduced the total number of *Phlebotomus sergenti* indoors and CL incidence one year post-intervention.^10^

Only a few small-scale research studies on insecticide-treated nets using CITNs for CL, have been published^4^ ^,^ ^11^ ^,^ ^12^ ^,^ ^13^ ^,^ ^14^ with contradictory results. In Colombia, lambda-cyhalothrin CITNs significantly reduced the indoor density of *L. trapidoi* and *L. gomezi* after net deployment in a study in the Department of Boyacá;^11^ although *L. longiflocosa* indoor densities did not decrease after lambda-cyhalothrin CITN deployment in the department of Huila, blood feeding success measured by the percentage of blood-fed females and number of fully-engorged female sand flies decreased.^4^ In Turkey and Syria, community studies with deltamethrin CITNs failed to reduce *P. sergenti* densities but showed a significant reduction in CL cases.^12^ ^,^ ^13^ These results are consistent with a household study in Afghanistan, which indicates a significant reduction in the incidence of CL due to permethrin CITNs.^14^

To improve the efficacy of CITNs and to convert untreated nets into LLINs, ‘dip-it-yourself’ (DIY) kits for deltamethrin (K-O Tab 1-2-3^®^) and lambda-cyhalothrin (ICON^®^ Maxx), as active ingredients, have been developed and tested. Two community studies in Bangladesh, India and Nepal showed a significant reduction in *P. argentipes* density due to deltamethrin treated CITNs, DIY CITNs and LLINs deployment; duration was highest for LLINs, followed by DIY CITNs and shortest for CITNs.^15^ ^,^ ^16^

Although, there is some evidence that vector control methods are effective in reducing sand fly densities, there are few well-designed, rigorous large-scale trials of sufficient duration, particularly for the impact of LLINs, on the effects of these control methods on sand fly density and leishmaniasis transmission. To address this gap, we evaluated the entomological impact of two LLINs, deltamethrin factory-treated nets and DIY lambda-cyhalothrin-treated net kits, for up to two years post-intervention in a large-scale trial. The effects on sand fly indoor density, feeding success (blood-fed and fully engorged females), parity and natural infection with *Leishmania* were evaluated. The residual effect of the LLINs and some acceptability indicators were also evaluated.

## MATERIALS AND METHODS

**Fig. 1:**
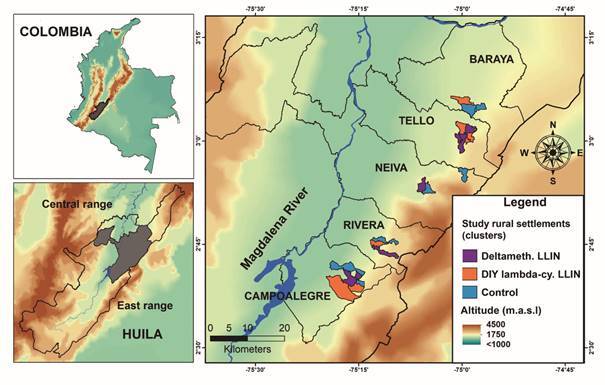
study area in five municipalities, on the sub-Andean region (east range) of Colombia, showing the participating 21 rural disperse settlements (clusters) where the field trial with long lasting insecticide-treated nets (LLINs) was carried out. Treatments (study arms): deltamethrin long lasting insecticide-treated nets (Deltameth. LLIN), dip it yourself lambda-cyhalothrin long lasting insecticide-treated nets dip it yourself (DIY) lambda-cy. LLIN and untreated nets (Control).

**Fig. 2:**
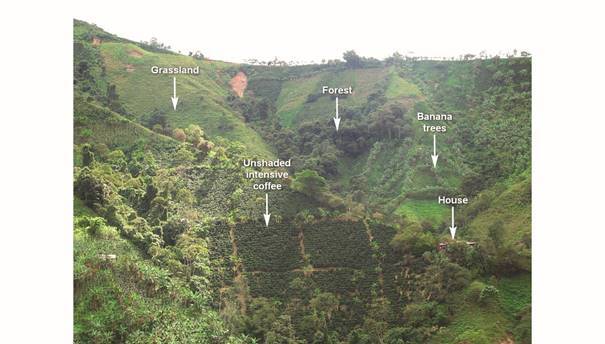
representative habitats of the dispersed rural settlements where the trial was carried out (Rural settlement of Arrayanal, Rivera Municipality, Department of Huila).

*Study area* - This study was conducted in an endemic/epidemic focus of CL located in the sub-Andean region of Colombia, surrounding the Magdalena River Valley in the Department of Huila (Colombia) (Fig. 1), that included five municipalities (Baraya, Tello, Neiva, Rivera and Campoalegre). Huila Department has reported two CL epidemics, one from 1993-1996, representing one of the largest ever reported in Colombia, with over 1,200 reported cases, and a 2003-2004 epidemic with 318 cases. In the last decade CL incidence has decreased to endemic levels of a median annual incidence of 22 cases.^17^ We conducted our trial, in 21 of 112 rural settlements dispersed across five municipalities (Fig. 1), selected based on their previous history of CL (at least one case from 2004 to 2009) and the appropriated security conditions for the research team. The annual rainfall pattern is bimodal in the region, with low rainfall occurring from December to February and June to September. According to the Holdridge life zones classification, the majority of the study settlements were located in the premontane moist forest (annual rainfall: 1000-2000 mm, temperature: 18-24ºC). Only fragments of the sub-Andean forest remain in the region, and land is primarily devoted to growing unshaded intensive coffee plantations (Fig. 2).

**Fig. 3:**
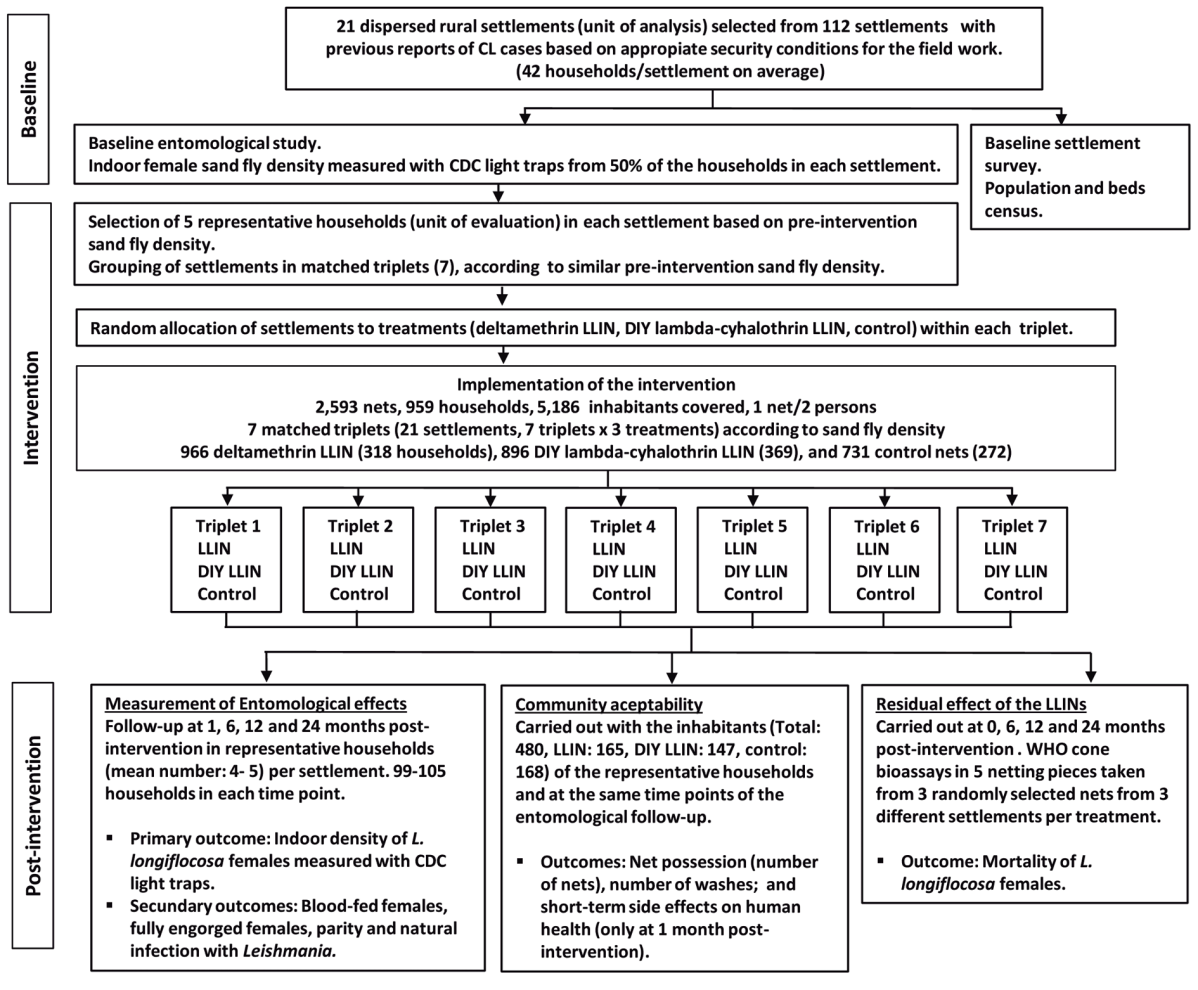
study design.

*Experimental design* - A matched-triplet cluster-randomised study at rural settlement level was used to evaluate the efficacy of two types of LLINs on indoor density, and other entomological variables, of the phlebotomine sand fly *L. longiflocosa* compared with a control. Twenty-one disperse rural settlements were distributed in seven matched triplets, within which sand fly collections were conducted at baseline (pre-intervention) and at 1, 6, 12, and 24 months post-intervention (Fig. 3).

*Baseline rural settlement survey* - We carried out baseline surveys between July and September 2010 to estimate the number of required nets needed for the intervention. All 959 households located in 21 rural settlements were visited by research team members to apply a questionnaire on the demographic characteristics (gender and age) of the residents and the number and size of used beds.

*Baseline entomological study* - Because of the high degree of spatial heterogeneity in sand fly densities, we carried out pre-intervention entomological surveys to characterise each settlement. We used the pre-intervention sand fly density to rank each settlement to blocks from highest to lowest for randomisation of the intervention within each block. Phlebotomine sand flies were collected simultaneously with the baseline rural settlement survey from approximately 50% of the households in each of the 21 settlements by trained research team members using CDC light traps (1 trap per night per household). Traps were placed in the bedroom where the highest number of occupants slept. Sampled households were selected based on the following criteria: (1) located within the altitudinal range of 1,400 - 1,800 m a.s.l. (where *L. longiflocosa* is more abundant), (2) inhabited for at least the month before the sampling and (3) a maximum of 90 min of foot travel time from the nearest road to the household.

*Selection of settlements and treatments allocation* - We ranked each selected settlement based on pre-intervention indoor density of *L. longiflocosa* females and block randomisation was carried out for each group of three settlements mostly closely matched on this parameter. Rural settlements within each triplet were randomised to one of the following three treatments using a function to generate random numbers in Excel (Microsoft): DIY lambda-cyhalothrin LLIN, deltamethrin LLIN, and control, untreated nets. The intervention was conducted in seven matched triplets (n = 7), including 21 rural settlements (seven allocated to the deltamethrin LLIN, seven allocated to the DIY lambda-cyhalothrin LLIN, and the remaining seven served as control) (Fig. 3).

*Unit of analysis and unit of evaluation* - Each cluster (one 21 dispersed rural settlement selected) had a mean of 42 houses. Within each cluster, we selected the five households consistent with previous entomological intervention trials,^7^ ^,^ ^18^ with the highest pre-intervention *L. longiflocosa* densities as unit of evaluation for post-intervention monitoring. Therefore, up to 105 household surveys were carried out during each post-intervention survey across the 21 settlements. During 1-month post-intervention survey 103 households were sampled because CDC light traps from two households (DIY lambda-cyhalothrin LLIN treatment) failed to operate. During 24-months post-intervention survey only 99 households were sampled because residents of six households (1 DIY lambda-cyhalothrin LLIN and 5 deltamethrin LLIN treatments) reported not using the nets.

*Treatments* - The characteristics of the three net interventions were: (1) DIY lambda-cyhalothrin LLIN, 60 mg/m^2^ (ICON^®^ Maxx, Basel, Basel-Stadt, Switzerland). This LLIN were obtained by dipping (dip-it-yourself) new untreated nets in an slow release capsule suspension formulation containing lambda-cyhalothrin combined with a polymer-binding agent in batches of ten nets by members of the research team following the protocol of Rozendaal;^19^ (2) Deltamethrin LLIN, 55 mg/m^2^ (PermaNet^®^ 2.0, Lausanne, Vaud, Switzerland). Nets factory coated with deltamethrin were acquired from private providers; and (3) Control. Untreated nets were also acquired from private providers. All nets were made of polyester which was white in colour. The nets in the deltamethrin LLIN treatment had a mesh size of 2.0 mm and 24 perforations/cm^2^. The nets in the other two treatments had a mesh size of 0.5 mm and 210 perforations/cm^2^ (they were considered sand fly proof bednets regardless of their treatment, because their small mesh size prevents sand flies from entering).

*Entomological endpoints measured* - The primary outcome (response variable) was the mean indoor density of *L. longiflocosa* females per CDC light trap (household) per night per settlement or cluster (n = 5 households). Secondary response variables were: (i) the percentage of blood-fed females [(number of females with any blood observed in the abdomen/total females collected) x 100]; (ii) the percentage of fully engorged females [(number of females with > 75% of their abdomen filled with blood /total blood-fed females) x 100]; (iii) the percentage of parous females [(number of females with ≥ 1 dilatation or presence of follicular sacs /total females collected) x 100]; and (iv) the natural infection with *Leishmania*.

*Sample size* - We estimated sample size based on the vector densities using the method for pair matched randomised controlled trials described by Hayes and Bennett.^20^ We expected to show a 50% reduction in *L. longiflocosa* density indoors in either of the two LLINs treatments compared with control. Based on the baseline entomological study data, we assumed a mean density of 11.6 sand flies/CDC light trap/night in the control rural settlements, a standard deviation of 3.76, a coefficient of variation (K) between settlements (clusters) of 0.28 and a cluster size of five households. Under those assumptions, we anticipated a power of 90% with a significance level of 5%. The minimum sample size calculated was seven rural settlements per treatment (intervention arm), with a total of 21 rural settlements (three treatments x seven replicates).

*Intervention deployment* - The three treatments: DIY lambda-cyhalothrin LLIN, deltamethrin LLIN, and control (untreated nets) were delivered free of charge to the households of the study settlements by the research team from late July to August 2012 (Fig. 1). All beds per settlement were covered with nets of the assigned treatment. The head of the household was advised to hang the nets at a rainfall-protected site outside the house the night before the start of their net use. This advice originated from the recommendations given by one of the LLIN manufacturers to reduce the short-term adverse effects of new nets on the health of the users. It was also recommended to all inhabitants of the participating settlements to use the nets on a daily basis and to wash them as seldom as possible. For washing, the following recommendations were given: (1) do not leave to soak, (2) do not use bleach, and (3) dry in the shade. The numbers of washes and the dates of the washes were recorded by the householders on a form that was distributed when the nets were delivered.

*Post-intervention evaluation* - Post-intervention evaluations were performed August 2012 to August 2014, at 1 month, 6 months, 12 months, and 24 months after net deployment. All the study phases (including the pre-intervention) were performed during months with low rainfall (January-March and July-September) when *L. longiflocosa* (known as a dry season species) is present at acceptable densities. Performing sampling activities during other months is hampered because *L. longiflocosa* populations decrease dramatically. The two-year delay between the pre-intervention survey and the intervention was due to an unexpected long and strong raining period, La Niña event, which lasted from late 2010 to 2011 and, forcing us to postpone the intervention to July 2012. The indoor density of *L. longiflocosa* per settlement was recorded using the CDC light traps as described for the baseline entomological study. Within each matched-triplet of settlements, the sand fly sampling was simultaneously or nearly simultaneously (no more than one week later).

*Sand fly processing and identification* - During the baseline sampling, the majority (90%) of the entomological samples, were preserved in ethanol. The remaining samples were kept in 10% dimethyl sulfoxide and frozen in liquid nitrogen. Only samples with the highest numbers of sand flies were frozen because logistical limitations hampered the freezing of all the samples. By contrast, all the post-intervention samples were frozen in liquid nitrogen. Subsequently, the samples were taken to the Laboratory of Entomology at the National Institute of Health in Bogotá D.C. Initially, for each frozen female that was previously immersed in a drop of phosphate-buffered saline pH 7.2, the blood-fed (female with any amount of blood) and blood amount (partially fed: 1- 75% of the abdomen engorged with blood; and fully engorged: > 75% of the abdomen engorged with blood) condition was recorded. After that, each female was dissected to remove the ovaries and alimentary tract. The alimentary tract was examined for the presence of motile promastigotes. Eppendorf microtubes containing phosphate-buffered saline 10% were available to preserve the gut contents of promastigote-positive sand flies at -20ºC before parasite identification by molecular methods. The ovarioles that detached in the ovaries were observed using phase contrast microscopy at 400x magnification to determine the sand fly parity status. The females were classified as nulliparous if the membrane of the terminal ovariole stalks was intact, and parous if the membrane was distended (large internal space, at least three times the length of the ovariole), which was indicative of a recent oviposition, or if at least one follicular dilatation was observed.^21^ Finally, the species identification was confirmed by examining the spermathecae in a drop of saturated phenol. Sand flies preserved in ethanol and males in liquid nitrogen were cleared in hot potassium hydroxide 10% and transferred to a drop of saturated phenol. All the sand fly dissections and identifications were performed by members of the research team, experts in medical entomology, using the taxonomic keys of Young and Duncan.^22^

*Residual lethal effect of the LLINs* - The lethal residual effect of the LLINs against *L. longiflocosa* was evaluated, under field conditions, using the Word Health Organization’s (WHO) cone bioassays, at 0 (unused new nets), 6, 12 and 24 months post-intervention. For each bioassay, groups of 15-20 field-caught, unfed *L. longiflocosa* females were exposed simultaneously to a piece of netting (30 x 30 cm) taken from a deltamethrin LLIN, DIY lambda-cyhalothrin LLIN and an untreated net (control) for 3 min. After exposure, the surviving sand flies were maintained in containers for 24 h, and their mortality was recorded. The sand flies were preserved and identified as described for the baseline entomological study. The selection of the netting pieces and nets was made as follows: one netting piece was randomly obtained from the side panels of three different nets and replaced by another netting piece of comparable size for each type of treatment (total = 9 nets). Rural settlements, households, and the sampled nets within a household were also randomly selected. At each time point the three netting pieces for each treatment were tested. Because the bioassay was replicated five times, two netting pieces from each treatment had to be tested twice. The same nets were used to obtain the netting pieces tested at each of the four post-intervention time point. The bioassays were carried out within the peridomicile of a house in the study area from 19:00 to 24:00 h.

*Community acceptability: usage and washing of nets* - Community acceptability of the LLINs was evaluated in a structured questionnaire survey applied within the same households (105 out of 959 participating households) and at the same four time points of the post-intervention entomological study. The researchers administered the questionnaire during house visits. The respondents were adult household members who answered the questions by consensus. The topics included in the questionnaire were about net possession (number of nets), net use, frequency of use, reasons for no use, when applicable, and number of washes. The net use was estimated initially by taking the raw percentage of nets in use (U_r_), as calculated as follows:^6^

$$U_{r}=\;\;\frac{number\;of\;mounted\;nets\;above\;the\;beds}{number\;of\;delivered\;nets}\;x\;100$$

To account for the lost nets, the majority due to the movement of household members (who carried their nets) outside the study area, the adjusted percentage of nets in use (U_a_) was also calculated as follows:

$$U_{a}=\;\;\frac{number\;of\;mounted\;nets\;above\;the\;beds}{\left(number\;of\;delivered\;nets-number\;of\;nets\;lost\;due\;to\;movements\;of\;persons\;outside\;the\;study\;area\right)}\;x\;100$$

The interviewers visually confirmed the numbers of mounted and dismounted nets.

*Adverse effects on human health* - In addition to inquiries on net use, an evaluation of the short-term side effects from the LLINs on human health was performed at one month post-intervention. Previous studies have shown that the side effects of insecticide-treated nets last no more than two weeks after net deployment.^23^ The potential side effects attributed to the LLINs were investigated by age and gender for each household member by recording their symptoms, starting date, and duration of the symptoms. The proportion of households reporting adverse effects during the first month after net delivery was calculated.

*Statistical analysis* - To ensure data quality, a data management system was implemented. To reduce possible acquisition, processing and analysis errors, the following measures were taken: (i) data were captured in the field using forms and questionnaires which were reviewed by a supervisor the same day; (ii) data bases were generated using the software Epi Info V 3.5.4 within which interactive checking was performed; (iii) each database was checked manually and for consistency to identify transcription errors; and (iv) during the analysis, box and whisker plots on the continuous and discrete variables were used to detect and correct outliers.

Considering the overwhelming dominance of *L. longiflocosa* females, which comprised over 82% of all the captures, the analysis was performed only on this species and sex. The density of *L. longiflocosa* females (primary response variable) is presented by treatment as the Williams geometric mean (GM_w_) for the pre-intervention (baseline sampling), at each time point post-intervention and for the total post-intervention (an average of all the time points) with their respective 95% confidence intervals. Initially, mean density of *L. longiflocosa* per trap (house) for each settlement were compared for the two LLINs treatments and the control at pre-intervention using Generalized Linear Models (GLM). This comparison allowed us to confirm the homogeneity in the primary response variable between treatments before the intervention. Then, the same comparison to evaluate the effect of the LLINs on the sand fly density was performed at each time point post-intervention and for the total post-intervention. The GLM analysis was performed assuming a Poisson distribution of errors. The model for the analysis of the total intervention effects from the LLINs included the arithmetic mean of the mean number of sand fly females for each settlement at each of the time points as the response variable and the net treatment as the primary explanatory variable. The models for each time point post-intervention include the same variables, except that sand fly density was the arithmetic mean of the number of female sand flies per trap for each settlement. The matched (block) effect, mean altitude and squared mean altitude were also included according to the significance in each model. Outlier data (1 and 2) were present in the models at 6 months and 12 months. Removing these outliners improved the fit of the models without any effect on the trends in the significance test. With these findings in mind, we showed only the values of the significance tests for the models from which the outliers were removed. Reduction in density of female sand flies previous confirmation of significant statistical differences between a LLIN and the control, was estimated using the coefficients of the models.

The percentages of blood-fed females, fully engorged females and parity are shown as the mean percentages by treatment for the pre-intervention, at each time point post-intervention and the total post-intervention with their respective 95% confidence intervals. Analyses of these variables using GLM was not possible because the models could not be fitted to the data. Initially, percentages of *L. longiflocosa* for each variable were compared for the two LLINs treatments and the control at pre-intervention using chi-square test (*X* ^2^). Afterwards, an evaluation of the effect of the LLINs on these variables was performed by comparing the ratio of the post-intervention percentage:pre-intervention percentage for each of the two LLINs treatments with the equivalent ratio for the control. These ratios were compared using the Kruskal Wallis test. When the test was significant, a pair comparison between treatments followed using the Mann-Whitney test.

The adjusted percentage of net use by treatment was analysed using the chi squared test or Fisher’s exact test when appropriate. The percentage of households reporting side effects on human health during the first month after the nets were delivered was compared among the net treatments using the chi squared test. All the analyses were performed using Stata 11 software.

*Ethical considerations* - This study was approved by the Ethics Committee of the National Institute of Health of Colombia (approved by agreement no. 5, Jun 25, 2009). It was explained to the householders of the participating households that some short-term, mild side effects (e.g., sneezing, nasal or skin irritation) could occur during the first weeks of their net use. An informed consent form was voluntarily signed by the head of each household included in the study.

## RESULTS

Entomological effects of the LLINs

*Baseline settlement survey and entomological study* - The census performed at the household level showed that the 21 rural settlements consisted of 959 houses and 5,186 inhabitants with a mean of 5.4 persons per household. A total of 2,593 beds were found in use.

The baseline entomological sampling included 52.3% (502 houses) of all the houses. A total of 17,833 sand flies belonging to eleven sand fly species of the *Lutzomyia* genus were caught inside the sampled houses. *L. longiflocosa* was the most dominant species, accounting for 92.4% (females: 14,606, and males: 1,874) of all the captures. The second and third most common species were *L. trinidadensis* (4.6%) and *L. longipalpis* (1.7%). An additional eight species comprised ≤ 0.6% per species included: *L. atroclavata*, *L. nuneztovari*, *L. dubitans*, *L. columbiana*, *L. carpenteri*, *L. oresbia*, *L. punctigeniculata*, and an unidentified species of the *Helcocyrtomyia* subgenus*.* The following analyses are limited to female *L. longiflocosa*, because they represent 82% of sand flies observed.

*Effect of the LLINs on the indoor sand fly density* - A total 2,593 new nets were deployed in the 959 households (5,186 inhabitants) within 21 settlements comprising the study. In each household, 100% of beds were covered with nets of the assigned treatment with a mean number of 2.7 nets per household and one net for every two persons (Fig. 3).

As expected, the pre-intervention GM_W_ female density was similar without significant differences across the three treatments, ranging from 17.8 to 18.5 females/light trap/night (*F*_(2,11)_ = 1.013, p = 0.394) (Table I). A total of 5,653 *L. longiflocosa* females were collected over 377 catches inside houses during the two years of post-intervention. There was a statistically significant difference between the treatments (*F*_(2,11)_ = 45.1138, p < 0.001). The post-intervention density of *L. longiflocosa* females caught inside the houses was significantly lower in the deltamethrin LLIN treatment, 4.7 (3.7-5.9, total = 1,052) females/light trap/night, and for the DIY lambda-cyhalothrin LLIN, 5.6 (4.6-6.7, total = 1,152) females/light trap/night, in comparison with the control, 15.5 (12.6-19.2, total = 3,449) females/light trap/night (*z* = -8.82, p < 0.001; and *z* = -8.80, p < 0.001, respectively) (Table I). These findings indicated 75.6% and 73.9% reductions for the DIY lambda-cyhalothrin LLIN and deltamethrin LLIN treatments, respectively. No significant differences in the densities of the females were found between the two LLIN treatments (*z* = 0.37, p = 0.711).

**TABLE I:** Density of *Lutzomyia longiflocosa* indoor by treatment at pre-intervention and at each time point post-intervention

Time point or triplet	Control (untreated nets)			DIY lambda-cyhalothrin LLIN			Deltamethrin LLIN			Statistical significance	
	No. females	GM_W_^*a*^	(CI^*b*^ )	No. females	GM_W_	(CI)	No. females	GM_W_	(CI)		
Pre-inter^*c*^ (triplets)											
1	895	152.6	(27.5 - 827.6)	831	159.4	(104.9 - 242.0)	804	155.3	(108.6- 221.7)		
2	274	42.8	(16.6 - 108.1)	296	50.6	(23.3 - 108.3)	430	91.2	(31.4 - 261.1)		
3	185	26.0	(7.4 - 85.7)	304	23.0	(2.1 - 183.8)	183	34.0	(19.9 - 57.6)		
4	51	12.6	(9.7 - 16.3)	93	13.7	(3.8 - 44.4)	58	10.1	(4.6 - 21.2)		
5	59	7.8	(1.4 - 31.0)	37	6.6	(3.0 - 13.4)	169	10.3	(0.4 - 88.4)		
6	79	6.4	(0.4 - 92.7)	29	4.9	(1.8 - 11.4)	30	5.8	(3.8 - 8.5)		
7	35	5.2	( 1.4 - 15.2)	36	6.4	(3.0 - 12.9)	5	1.2	(0.6 - 2.1)		
Total	1578	17.8	(10.5 - 29.9)	1626	18.2	(11.0 - 29.7)	1679	18.5	(10.3 - 32.7)	*F*_(2,11)_ = 1.01	p = 0.394
Post-inter^*d*^ (time point)											
1	338	6.8	(4.2 - 10.5)	108	2.3	(1.5 - 3.4)	91	1.5	(0.9 - 2.4)	*F*_(2,12)_ = 44.48	p < 0.001
% Reduction					68.9	(50.0 - 80.7)		73.2	(55.7 - 83.8)		
6	1574	26.4	(16.4 - 41.9)	410	7.0	(4.5 - 10.6)	444	8.0	(4.9 - 12.8)	*F*_(2,10)_ = 24.65	p < 0.001
% Reduction					67.0	(52.1 - 77.3)		62.1	(47.1 - 72.9)		
12	792	17.9	(12.4 - 25.7)	309	6.9	(4.9 - 9.6)	302	6.0	(4.0 - 8.9)	*F*_(4,9)_ = 26.82	p < 0.001
% Reduction					61.7	(40.6 - 75.4)		62.6	(44.1 - 75.0)		
24	745	18.1	(13.1 - 24.8)	325	7.5	(5.6 - 9.9)	215	6.0	(4.0 - 8.7)	*F*_(2,12)_ = 15.27	p < 0.001
% Reduction					60.3	(39.9 - 73.7)		63.6	(44.2 - 76.2)		
Total	3449	15.5	(12.6 - 19.2)	1152	5.6	(4.6 - 6.7)	1052	4.7	(3.7 - 5.9)	*F*_(2,11)_ = 45.11	p < 0.001
% Reduction					75.6	(64.3 - 83.7)		73.9	(62.5 - 81.8)		

*a*: geometric mean of Williams of females/CDC light trap/night; *b*: 95% confidence interval; *c*: pre-intervention; *d*: post-intervention.

**Fig. 4:**
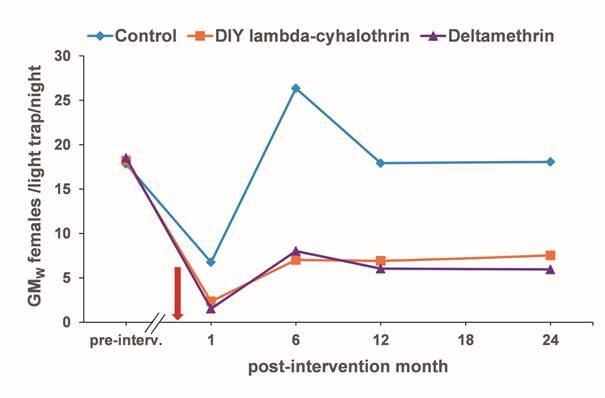
effect of the long lasting insecticide-treated nets (LLINs) on *Lutzomyia longiflocosa* indoor density, geometric mean of Williams (GM_w_) of females/CDC light trap/night. Pre-intervention (pre-interv.) survey was carried out from July to September 2010 and deployment of nets, showed by the red arrow, from July to August 2012.

For each post-intervention survey (1, 6, 12 and 24 months), the density of *L. longiflocosa* females inside the houses under both LLIN treatments was significantly lower compared with the control (Table I, Fig. 4). The percent reduction in *L. longiflocosa* females for deltamethrin LLIN ranged between 62.1% and 73.2%, and for DIY lambda-cyhalothrin LLIN, between 60.3% and 68.9%. No significant differences in the female densities were found between the two LLIN treatments for any of the time points (statistical tests not shown).

**Fig. 5:**
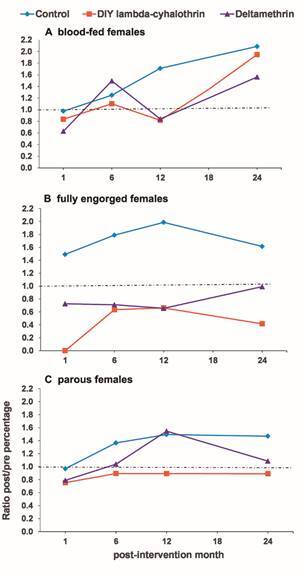
effect of the long lasting insecticide-treated nets (LLINs) on *Lutzomyia longiflocosa* indoor average ratio of post-intervention/pre-intervention percentages (post/pre) of: (A) blood-fed females, (B) fully engorged females, and (C) parous females.

*Effect of the LLINs on blood-feeding success* - The percentages of blood-fed females and fully engorged females for the pre-intervention and post-intervention phases of the study are shown in Table II. While the female density pre-intervention was similar for all the treatments (both LLINs and the control) as a result of the matching of settlements in triplets by the sand fly density, the pre-intervention percentage of blood-fed and fully engorged females were unbalanced. There were statistically significant differences between the treatments in terms of the percentages of blood-fed females, with a range of 5.5-12.2% (χ^2^ = 46.172, df = 2, p < 0.001), and fully engorged females, with a range of 45.5-75.6% (χ^2^ = 20.062, df = 2, p < 0.001) (Table II). Although the overall post-intervention mean ratio of the post-intervention:pre-intervention percentage of blood-fed females was slightly reduced in the deltamethrin LLIN and DIY lambda-cyhalothrin LLIN treatments, at 1.2 (15.2/12.2, and 6.9/5.5, respectively) each, compared to the control, 1.5 (12.8/8.5), there was not a significant difference between the treatments (Kruskal Wallis test, χ^2^ = 1.499, df = 2, p = 0.473). At each of the four post-intervention time points, the ratio of the post-intervention:pre-intervention percentage for blood-fed females followed the same overall post-intervention pattern (Fig. 5A), except at the six-month time point when the ratio for the deltamethrin LLIN was higher compared with the control. Nevertheless, there were no significant differences between treatments (statistical tests not showed).

**TABLE II:** Effects of the long lasting insecticide-treated nets (LLINs) on blood-fed females, fully engorged females and parity of *Lutzomyia longiflocosa* at each of the sampling time points collected with CDC light traps indoors

Variable	Sampling		Control (untreated nets)			DIY lambda-cyhalothrin LLIN			Deltamethrin LLIN		
			No. females	Percentage	(CI^*d*^ )	No. females	Percentage	(CI)	No. females	Percentage	(CI)
Blood-fed females^*a*^	Pre-intervention		1578	8.5	(7.2 - 9.9)	1626	5.5	(4.5 - 6.8)	1679	12.2	(10.7 -13.9)
	Post-intervention month										
		1	338	8.3	(5.6 -11.7)	108	4.6	(1.5 - 10.5)	91	7.7	(3.1 - 15.2)
		6	1574	10.6	(9.1 - 12.2)	410	6.1	(3.9 - 8.9)	444	18.2	(14.8 - 22.1)
		12	792	14.5	(12.1 - 17.2)	309	4.5	(2.5 - 7.5)	302	10.3	(7.1 - 14.2)
		24	745	17.7	(15.0 - 20.6)	325	10.8	(7.6 - 14.7)	215	19.1	(14.0 - 24.9)
		Total	3449	12.8	(11.7 - 13.9)	1152	6.9	(5.5 - 8.5)	1052	15.2	(13.1 - 17.5)
Fully engorged females^*b*^	Pre-intervention		134	45.5	(36.9 - 55.3)	90	75.6	(65.4 - 84.0)	205	59.0	(51.9 - 65.8)
	Post-intervention month										
		1	28	67.9	(47.6 - 84.1)	5	0		7	42.9	(9.9 - 81.6)
		6	167	81.4	(74.7 - 87.0)	25	48.0	(27.8 - 68.7)	81	42.0	(31.1 - 53.5)
		12	115	90.4	(83.5 - 95.1)	14	50.0	(23.0 - 76.9)	31	38.7	(21.8 - 57.8)
		24	132	73.5	(65.1 - 80.8)	35	31.4	(16.8 - 49.3)	41	58.5	(42.1 - 73.7)
		Total	442	80.5	(76.5 - 84.1)	79	38.0	(27.3 - 49.6)	160	45.6	(37.7 - 53.7)
Parity^*c*^	Pre-intervention		874	18.0	(15.5 - 20.7)	683	22.1	(19.0 - 25.4)	758	16.7	(14.2 - 19.6)
	Post-intervention month																			
		1	338	17.5	(13.6 - 21.9)	108	17	(10.2 - 25.0)	91	13.2	(7.0 - 21.9)
		6	1574	24.6	(22.5 -26.8)	410	19.8	(16.0 - 23.9)	444	17.3	(13.9 - 21.2)
		12	792	26.9	(23.8 - 30.1)	309	19.7	(15.4 - 24.6)	302	25.8	(20.9 - 31.1)
		24	745	26.4	(23.2 - 29.8)	325	19.7	(15.5 - 24.4)	215	18.1	(13.2 - 23.9)
		Total	3449	24.8	(23.4 - 26.3)	1152	19.4	(17.2 - 21.8)	1052	19.6	(17.2 - 22.1)

*a*: blood-fed females percentage (number of blood-fed females/total of females) *100; *b*: fully engorged females percentage (number of females > 75% of abdomen filled with blood/number of blood-fed females) *100; *c*: parity percentage (number of parous females/total of dissected females) *100. *d*: 95% confidence interval.

However, there was a significant difference between the treatments in the overall post-intervention mean ratio of the post-intervention:pre-intervention percentage for fully engorged females (Kruskal Wallis test, χ^2^ = 11.099, df = 2, p = 0.004). The ratio was significantly lower, 0.5 (38.0/75.6) and 0.8 (45.6/59.0), in the DIY lambda-cyhalothrin LLIN and the deltamethrin LLIN treatments, respectively, compared with the control, 1.8 (80.5/45.5) (Mann-Whitney test, z = 2.882, p = 0.004, and z = 2.722, p = 0.007, respectively). There was no significant difference between the two LLINs (Mann-Whitney test, z = -0.801, p = 0.423). At each of the four post-intervention time points, the post-intervention:pre-intervention percentage ratio for the fully engorged females followed the same overall post-intervention pattern (Fig. 5B). The ratios of the two LLINs were significantly lower compared with the ratio of the control (statistical tests not shown), with an exception at the twelve month time point at which the ratio for the DIY lambda-cyhalothrin LLIN had borderline significance compared with the control. Again, there were not significant differences between the ratios of the two LLIN treatments (statistical tests not shown).

*Effect of the LLINs on parity* - The parity percentage was unbalanced pre-intervention, as occurred with the trophic status. The percentage of parity ranged between 16.7% and 22.2% with significant differences between treatments (χ^2^ = 7.377, df = 2, p < 0.025) (Table II). The overall post-intervention mean ratio for the post-intervention:pre-intervention percentage of parous females was apparently reduced at 0.9 (19.4/22.1) and 1.2 (19.6/16.7) in the DIY lambda-cyhalothrin LLIN and the deltamethrin LLIN treatments, respectively, compared with the control, 1.4 (24.8/18.0). Nevertheless, there was not a statistically significant difference (Kruskal Wallis, χ^2^ = 1.059, df = 2, p = 0.589). At the majority of the four time points post-intervention, the post-intervention:pre-intervention percentage ratio of parity followed the same overall post-intervention pattern (Fig. 5C). There were not significant differences between the treatments at each of the four time points post-intervention (statistical tests not shown).

*Detection of sand flies infected with Leishmania* - Throughout the study, a total of 9,799 (pre-intervention = 4,146; post-intervention = 5,653) *L. longiflocosa* females were examined to detect *Leishmania* parasites. *Leishmania* infected sand flies were not detected under the described methodology.

**Fig. 6:**
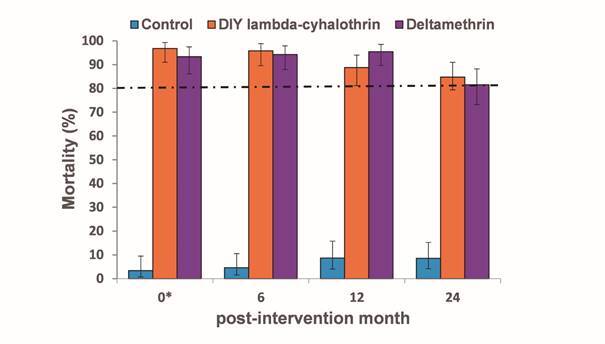
effect of the long lasting insecticide-treated nets (LLINs) used in field on 24 h post-exposure mortality of *Lutzomyia longiflocosa* females (n = 5; total number of females per treatment and time point = 89 - 117). Error bars show 95% confidence intervals. The dotted line shows the mortality threshold for high residual effect according to World Health Organization. *: new nets.

*Residual effect of the LLINs* - A total of 20 bioassays (five replicates x four time points) were performed on 1,238 field-caught *L. longiflocosa* females using the WHO contact bioassay cones. The mean temperature and relative humidity during the exposure were 20.3ºC (min = 18ºC, max = 22.8ºC) and 72.4% (min = 58.3%, max = 92.9%), respectively. Residents of the surveyed in the acceptability study rarely washed the test nets. At 12 and 24 months post-intervention, the median numbers of washes were one and two, respectively. The mortality at 24 h postexposure for both LLINs remained at over 80% up to 24 months post-intervention (Fig. 6). Mortality however, dropped significantly with time, in the DIY lambda-cyhalothrin LLIN post-intervention mortality was > 95%, 88.8% and 84.8% at 6, 12 and 24 months, respectively (χ^2^ linear trend = 11.550, df = 1, p < 0.001). Similarly, for the deltamethrin LLIN treatment mortality was > 93% up to 12 months post-intervention and 81.6% at 24 months post-intervention (χ^2^ linear trend = 11.335, df = 3, p = < 0.001). There were not significant differences between the two LLINs at each of the time points (data not shown). The mortality in the control treatment remained low (range 3.4-8.6%) for each of the time points.

Community acceptability

*Usage and washing of LLINs* - The adjusted percentage of net use remained high (96.8-100%) up to 12 months post-intervention for all treatments (Table III). At 24 months post-intervention, the adjusted percentage of net use remained high for the DIY lambda-cyhalothrin LLIN (94.1%) and the control (100%), but it dropped to 80% for the deltamethrin LLIN, with significant differences between treatments (χ^2^= 26.063, df = 2, p < 0.001). The adjusted percentage of net use in the control treatment was significantly higher compared with the DIY lambda-cyhalothrin LLIN (Fisher’s exact test = 0.018) and the deltamethrin LLIN (χ^2^ = 22.510, df = 1, p < 0.001). The adjusted percentage of net use in the deltamethrin LLIN treatment was significantly lower compared with that of the DIY lambda-cyhalothrin LLIN (χ^2^ = 7.632, df = 1, p = 0.006). The primary cause of not using the nets for both LLINs and the control for each time point post-intervention was user absence. Excluding user absence, taking into account in the adjusted percentage of net use, at 24 months post-intervention the main reason of not using the nets were the perception that “mantablanca” (local name given to the phlebotomine sand flies) passed through the nets (n = 13) or because nets suffered severe physical damage (n = 5) reported for the deltamethrin LLIN. Severe physical damage to the nets (n = 3) and undefined reasons (n = 2) were reported for the DIY lambda-cyhalothrin LLIN (Table III). With respect to the frequency of net use by the inhabitants, most, 93.3% (98/105), of the interviewees reported using the nets on a daily basis. A low number, 6.7% (7/105) of the interviewed individuals used the nets only during the nights when they perceived sand fly activity. This pattern remained over time regardless of the post-intervention time point. According to the records on washing frequency, the inhabitants of the households of the post-intervention entomological study washed their nets few times during the two years of the study, in accordance with the recommendation given by the research team, without significant differences between treatments (data not shown). At 6, 12 and 24 months post-intervention, the median numbers of washes were the same for each of the three types of nets: 1 (percentile_25_ = 1, percentile_75_ = 2), 2 (1-3), and 3 (2-5), respectively.

**TABLE III:** Percentage of nets in use by treatment at each of the time points post-intervention in representative houses for each settlement where entomological follow-up of the long lasting insecticide-treated nets (LLINs) trial was carried out

Post-intervention month	Control (untreated nets) (35)^*a*^						DIY lambda-cyhalothrin LLIN (35)						Deltamethrin LLIN (35)					
	No. nets						No. nets						No. nets					
	del.^*b*^	In use	not in use due to		percentage of net use		del.	in use	not in use due to		percentage of net use		del.	In use	not in use due to		percentage of net use	
			user absent	other reason	Ur^*c*^	Ua^*d*^			user absent	other reason	Ur	Ua			user absent	other reason	Ur	Ua
1	127	96	31	0	75.6	100	102	77	25	0	75.5	100	113	85	28	0	75.2	100
6	127	94	33	0	74.0	100	102	82	19	1	80.4	98.8	113	94	19	0	83.2	100
12	127	103	24	0	81.1	100	102	82	20	0	80.4	100	115	92	20	3	79.1	96.8
24	129	102	27	0	79.1	100	102	80	17	5	78.4	94.1	115	72	25	18	62.6	80.0

*a*: number of households; *b*: number of delivered nets; *c*: raw percentage of nets in use (number of mounted nets above the sleeping area/ number of delivered nets) *100; *d*: adjusted percentage of nets in use number of mounted nets above the sleeping area/ (number of delivered nets - number of nets lost due to movement of persons outside the study area) *100. DIY: dip it yourself.

*Adverse effects on human health* - Short-term side effects from the LLINs on human health were only reported by 4.2% (20/480) of the household members. Overall, the percentages of adverse effects were 2.0% (3/147) in the DIY lambda-cyhalothrin LLIN treatment, 4.2% (7/165) in the deltamethrin LLIN treatment, and unexpectedly, 5.9% (10/168) in the control (Table IV). Nevertheless, there were not significant differences between treatments (χ^2^ = 2.6479, p = 0.266). The most common side effect was nasal irritation, 45% (9/20), followed by dizziness and/or headache 25% (5/20). Other less common side effects were asphyxiation, itching and ocular irritation. All these side effects were reported only within the first week post-intervention, and they lasted for a median time of 1.5 days (percentile_25_ = 1; percentile_75_ = 2). The side effects were more frequently reported by women, 75% (15/20); and the majority, 80% (16/20), were reported by household adults aged 18-52 years old.

**TABLE IV:** Short term side effects of new long lasting insecticide treated nets (LLINs) reported during the first week of use by treatment in the inhabitants of representative households where entomological study was carried out (480 inhabitants in 105 households)

Side effects experienced by the family members	Control (untreated nets) (168)^*a*^				DIY lambda-cyhalothrin LLIN (147)				Deltamethrin LLIN (165)			
	n^*b*^	(m : w)^*c*^	%	Effect duration in days (min - max)	n	(m : w)	%	Effect duration in days (min - max)	n	(m : w)	%	Effect duration in days (min - max)
Dizziness and/or headache	4	(4 : 0)	40.0	(1 - 2)	0		0		1	(1 : 0)	14.3	1
Nasal irritation	4	(2 : 2)	40.0	(1 - 2)	1	(1 : 0)	33.3	1	4	(4 : 0)	57.1	(1 - 4)
Ocular irritation	1	(1 : 0)	10.0	8	0		0		0		0	
Asphyxiation	1	(1 : 0)	10.0	1	0		0		1	(0 : 1)	14.3	4
Itching	0		0		1	(0 : 1)	33.3	1	1	(1 : 0)	14.3	1
Itching and headache	0		0		1	(0 : 1)	33.3	1	0		0	
Total	10		5.9		3		2.0		7		4.2	

*a*: number of persons in each treatment; *b*: number of persons reporting side effects; *c*: m = men, w = women. DIY: dip it yourself.

## DISCUSSION

The results of the present study demonstrate that in dispersed rural settlements in the Colombian sub-Andean region, the deployment of deltamethrin and DIY lambda-cyhalothrin LLINs can lead to a significant reduction in the indoor density of *L. longiflocosa* (74-76%), and in the proportion of fully engorged females up to two years post-intervention. These results are supported by the findings on the residual effect of the LLINs and the community acceptability. Throughout the whole evaluation period the residual effect of both LLINs was above the efficacy threshold (mortality at 24 h > 80%). The percentages of net use (80-94%) and frequency of use on a daily basis (93%) were also high, the frequency of washing of nets was low (two washings per year) and there was a very low rate (2-4%) of mild short-term adverse effects on human health.

Entomological effects of the LLINs

*Effect of the LLINs on the indoor sand fly density* - The reduction in the indoor density of *L. longiflocosa* in the settlement treatments with both LLINs is probably explained by several effects previously detected with the same sand fly species and within the same study area: (i) The LLINs killed or incapacitated a significant proportion of sand flies that had enough contact with the LLINs to take in a lethal dose. This is probably the most important effect. The mortality of *L. longiflocosa* females at 24 h post-exposure in an experimental hut with deltamethrin LLINs or DIY lambda-cyhalothrin LLINs was very high, 82.6% and 78.8%, respectively, compared with 4.7% in the control.^24^ Comparable results were found in a field study with deltamethrin CITNs (25%) in Brazil, where the mortality of *L. longipalpis* at 24 h outside the net was 67.7%, compared with 0.4% in the control.^25^ (ii) The LLINs caused induced exophily. Hence, a proportion of females who entered the houses using LLINs were induced to leave the houses (due to an irritant and/or repellent effect) before they had the chance to enter the CDC light traps. A previous study with experimental huts found a significant increase in the proportion, 35.8% and 19.5%, respectively, of *L. longiflocosa* trapped in exit traps of the huts with DIY lambda-cyhalothrin LLIN and deltamethrin LLIN compared with control (6.3%).^24^ (iii) A deterrent effect cannot be discarded. In the same study with experimental huts, although not significant, there was an apparent reduction in the number of *L. longiflocosa* entering the huts, from 74 sand flies/night in the control to 52.6 and 43.1 sand flies/night in the deltamethrin LLIN huts and DIY lambda-cyhalothrin LLIN huts, respectively.^24^ The reduction in sand fly density collected by the CDC light traps must reflect a reduction in the *L. longiflocosa* biting rate on humans in both LLINs, as it has been shown that there were no differences in the ratio of CDC light trap:human bait when CITNs were compared with control houses for this sand fly species in the same study area.^4^

Few studies have been performed regarding the efficacy, either at the household level or in large-scale interventions at the community level, of LLINs against vectors of leishmaniases, and studies related to CL are scarce. Despite the short follow-up (less than a year), previous studies have found that LLINs caused a significant reduction in the density of sand flies indoors.^7^ ^,^ ^8^ ^,^ ^9^ ^,^ ^10^ ^,^ ^18^

Only two large-scale studies at the community level with LLINs, including permethrin LLINs, for CL have evaluated sand fly density. In the first study, carried out in Iran, the LLINs reduced the total number of *P. sergenti* females indoors, caught with CDC light traps, up to a few months post-intervention; unfortunately, the authors did not give information about the size of the reduction.^10^ In the second study, in Turkey, the LLINs did not reduce the post-intervention density of *P. tobi*, collected with CDC light traps and sticky traps after one year of follow-up.^26^ The results in the latter study were compromised because there was no replication, and the results on sand fly density are presented in a confusing manner. In contrast, several large-scale deltamethrin LLIN studies for VL have been carried out on the Indian subcontinent. In India and Nepal, an evaluation of deltamethrin LLINs, have shown a 25% reduction in the number of sand flies indoors (maximum pre-intervention density: 4-5 sand flies/trap/night) after a one-year follow up.^17^ The percentage of reduction in that study may have been underestimated because the LLINs were replaced by untreated nets during the sampling nights. In Bangladesh, LLINs reduced the indoor density of sand flies by 70-80% (pre-intervention density: 5 females/trap/night) up to five months post-intervention, and the density remained low up to 11 months.^9^ Two studies, one in Nepal and another in Bangladesh, India and Nepal, both with follow-up for five months post-intervention showed that LLINs caused a reduction of 61% and 44%, respectively, in the indoor density of sand flies (first study: control: 2.3 sand flies/trap/night; LLINs: 0.9 sand flies/trap/night; second study: control: 12.1 sand flies/trap/night; LLINs: 8.3 sand flies/trap/night).^8^ ^,^ ^18^ There are very few studies of LLINs at household level, and there have been no studies related to CL. A household study in India showed that up to two months post-intervention, neither deltamethrin nor permethrin LLINs caused a reduction in the female sand fly density indoors (pre-intervention density: 2-4 females/house).^27^ The aforementioned percentage reductions (from 25% to 80%) in the density of sand flies indoors, excepting two studies,^7^ ^,^ ^27^ is comparable to the percentage reduction of *L. longiflocosa* found in the present study by the deltamethrin LLINs, 74% (control: 16 sand flies/trap/night, LLIN: five sand flies/trap/night), with the difference that the present study demonstrated that the effect on sand fly density lasted up to two years post-intervention (at least twice the follow-up in previous studies). Therefore, the results of the present study support the long-lasting effect of deltamethrin LLIN.

The remarkably significant reduction in the density of sand flies caused by the DIY lambda-cyhalothrin LLIN, 76% (control: 16 sand flies/trap/night, LLINs: six sand flies/trap/night) up to two years post-intervention, was confirmed as well at 1, 6, and 12 months. In recent studies, although it has been found that DIY LLINs could reduce the indoor density of sand flies, the effect lasted for less than a year or the reduction was significant only for some of the sampled time points.^15^ ^,^ ^16^ In a large-scale study at the community level against VL in Bangladesh, India and Nepal, up to one year post-intervention, the DIY deltamethrin LLINs reduced the sand fly density of *P. argentipes* indoors, measured as the incidence rate ratio, only up to nine months post-intervention.^16^ In a similar study, with the same type of DIY LLIN plus a synergist applied in Bangladesh with a 1.8 years post-intervention follow-up period, the DIY LLINs caused a reduction in the density of *P. argentipes* indoors from 4% to 74% (pre-intervention density: 7-12 sand flies/trap/night), with significant reductions 5, 14, 15 and 18 months post-intervention.^15^ To our knowledge, the present study is the first large-scale trial in which DIY lambda-cyhalothrin LLINs have been evaluated against sand flies. Finally, the lack of significant differences in the density of females of *L. longiflocosa* indoors between the DIY lambda-cyhalothrin LLINs (six females/trap/night) and the deltamethrin LLINs (five females/trap/night) indicates that the DIY LLINs performed as well as the factory-treated LLINs, at least up to two years post-intervention.

*Effect of LLINs on blood-feeding success* - Neither deltamethrin LLINs nor DIY lamda-cyhalothrin LLINs affected the proportion of blood-fed females (females with any blood observed in the abdomen) of *L. longiflocosa*, as there were no significant differences in the ratios of the post-intervention:pre-intervention percentages of blood-fed females between treatments. This result could be explained by the low percentage of blood-fed females collected regardless of the treatment used during the study, ranging from 5.5% to 12.2% at the pre-intervention and from 4.5 to 19.1 at the post-intervention time points.

Although blood feeding success, represented by blood-fed females and/or fully engorged females, is negatively affected by CITNs,^6^ few field studies of LLINs for leishmaniasis control have measured this variable with poor results. Two evaluations of deltamethrin LLINs in Bangladesh^9^ and another in India and Nepal^7^ also recorded very low percentages of blood-fed females of *P. argentipes* over the course of the interventions precluding any analysis.

Both deltamethrin LLINs and DIY lambda-cyhalothrin LLINs caused a significant reduction in the ratios of the post-intervention:pre-intervention percentages of fully engorged *L. longiflocosa* females. Compared with the control, the percentage reduction in the post-intervention:pre-intervention percentage ratios of fully engorged females was 72.2% (1.3/1.8) and 55.5% (1/1.8) for the DIY lambda-cyhalothrin LLINs and deltamethrin LLINs, respectively. The reduction in fully engorged females might be explained by an effect of the insecticide on the feeding behavior of the female sand flies after they started to take the blood meal, which make females stop feeding and therefore, preventing some of them from obtaining a full blood meal. Females of many sand fly species need a single full blood meal for egg maturation and laying in each gonotrophic cycle. Thus, feeding success is related to fecundity (number of eggs laid per female) and population size and therefore the reduction in feeding success (percentage of fully engorge females), suggests that in large-scale trials with completed coverage with LLINs, a negative effect on the sand fly population of *L. longiflocosa* could be expected. The results of the present study are compatible with the data from the household study performed in the same sand fly species and within the same study area where lambda-cyhalothrin CITNs caused a significant reduction in the number of fully engorged females, from 13 sand flies/CDC trap/night in the control to 0.9 sand flies/CDC trap/night in the CITNs treatment.^6^ Finally, the lack of significant differences in the ratio post-intervention:pre-intervention percentage of fully engorged females of *L. longiflocosa* indoors between the DIY lambda-cyhalothrin LLIN and the deltamethrin LLIN gives additional support to the idea that the DIY LLINs performed as well as the factory-treated LLINs, at least up to two years post-intervention.

*Effect of the LLINs on parity* - The present study gives no evidence for an effect of either LLINs on the ratio of the post-intervention:pre-intervention percentage of parous females of *L. longiflocosa* indoors. The percentage of parous females ranged from 16.7% to 22.1% during pre-intervention collections and from 13.2% to 26.9% during post-intervention collections. The values of this variable in the present study are similar to the percentage of parous females recorded for *L. evansi*.^28^ In contrast, higher percentage of parous females, 38-64%, were reported for *L. longipalpis* at resting sites, including indoors.^29^ The lack of reduction in parity suggests that under the conditions of the present study, neither LLINs affected the population size (the number of individuals in the population inhabiting an area) of *L. longiflocosa*.

*Detection of sand flies infected with Leishmania* - Worldwide natural infection with *Leishmania* in sand flies is generally very low, and seldomly exceeds 2.0%.^30^ With respect to *L. longiflocosa* a study in the department of Tolima, in a region comparable to our study area in the department of Huila, natural infection with *Le. guyanensis* ranges from 0.7 to 2.7%.^5^ The failure of detection of *Leishmania* infection of the present study, in spite of the large number (near 10,000 females) of dissected sand flies, may have been due to the following two reasons: (i) sampling method was not designed to target parous females which are more likely to be infected, and (ii) the study was carried out during an endemic period, more than six years after the last epidemic.

Community acceptability

*Usage and washing of LLINs* - Percentage of net use dropped only up to 24 months post-intervention for both LLINs treatments, with a significant lower, 80%, adjusted percentage of net use for the deltamethrin LLIN followed by the lambda-cyhalothrin LLIN, 94%. The primary reason of no use of the deltamethrin LLIN given by the interviewed: perception that sand flies passed through the deltamethrin LLIN, is supported in the larger mesh size, 2.0 mm, of this net compare with the DIY lambda-cyhalothrin LLINs and the control nets, both with a mesh size of 0.5 mm. Even though, there was a significant reduction in the adjusted percentage of net use of the LLINs, it is considered high as well as the frequency of net use (> 93% for all treatments), which is comparable to other studies. In India and Nepal deltamethrin LLIN was evaluated against VL including promotion of the correct use of the LLIN, while in the control (untreated nets) this activity was no promoted.^10^ After two years of follow-up, 91% of the household members slept regularly under a LLIN. In contrast, in the control group only 70% of household members used a net at least once during the trial. The results indicate that all nets, DIY lambda-cyhalothrin LLIN, deltamethrin LLIN and untreated nets (control) had high acceptability by the community members. Nevertheless, use of the deltamethrin LLIN could be threatened due to its mesh size, which allows the sand flies to pass through.

*Adverse effects on human health* - The very low report of adverse side effects for the household members of the DIY lambda-cyhalothrin LLINs (2.0%) and deltamethrin LLINs (4.2%) treatments, reported only during the first week of net usage, was in general comparable with other studies. An study in Bangladesh, India and Nepal reported at least less than 5% adverse side effects on household members of deltamethrin treated nets used against leishmaniasis.^16^ The low percentage of side effects reported in the present study might be due in some extent to the reduction in emanations in the new LLINs, which, by recommendation of one of the LLINs manufacturers, were hanged outside each house the night before the start of use. The type of reported adverse side effect (nasal irritation, dizziness, headache, asphyxiation, itching and ocular irritation) caused by the DIY lambda-cyhalothrin LLIN and deltamethrin LLIN were similar to those reported in other studies.^16^ The unexpected report of side effects in the control treatment (untreated nets) might be explained because the householders knew some nets were insecticide-treated, although they were unaware of the type of treatment they received. Hence, some household members might have reported to feel side effects as they believed the net they received was insecticide-treated when actually they received an untreated net.

*Study limitations* - The study has some limitations that are worth noting. Although the study demonstrated that both LLINs caused a reduction in the density of sand flies indoors for a longer time than in any other previous study, it is necessary to perform additional research extending the follow up to three years post-intervention to fully confirm the long-lasting effect.^6^ With respect to community acceptability, it is important to highlight that the results represent only the human population that lives in the houses which were visited frequently by members of the research team who reminded them of the correct use of the nets. Therefore, the acceptability indicators measured in the present study could be overestimated.

In summary, our results provide compelling evidence on the high efficacy of LLINs against indoor sand fly vectors of CL in the sub-Andean region of Colombia. Both the deltamethrin LLINs and the DIY lambda-cyhalothrin LLINs caused a reduction of 74-76% in *L. longiflocosa* females indoors and in the percentage of fully engorged females (proxy for feeding success) up to two years post-intervention. Hence, it is expected that the deployment of these LLINs in large-scale interventions could have a significant impact on human exposure to sand fly bites and thereby on the reduction of the risk of transmission of CL in the sub-Andean region. Given that there is a proportion of sand flies that apparently are not impacted by the LLINs and given that the inhabitants of the houses could be exposed to some bites at dusk and dawn, before protected by the LLINs, future work should combine the LLINs with some supplementary control methods (e.g., curtains on heaves, windows and doors inside the house, and spatial repellents around the house). The high efficacy of the DIY lambda-cyhalothrin LLINs comparable to that of the factory-treated deltamethrin LLINs is an important finding. The DIY LLIN formulation may be used to convert untreated nets into a near-LLIN with the additional advantage that the physical features of the nets could be accommodated to the specific requirements of the region and community to be intervened. Our results provide evidence to policy makers that support the use of LLINs and DIY LLINs in vector control of CL in the Andean region.
